# Rapid Analysis of Pharmacology for Infectious Diseases

**DOI:** 10.2174/156802611795429130

**Published:** 2011-05

**Authors:** Andrew L Hopkins, G. Richard Bickerton, Ian M Carruthers, Stephen K Boyer, Harvey Rubin, John P Overington

**Affiliations:** 1 Division of Biological Chemistry and Drug Discovery, College of Life Sciences, University of Dundee, Dow StreetDundee, DD1 5EH, UK; 2 IBM, Almaden Research Center, 650 Harry Road, San Jose, California 95120-6099, USA; 3 Departments of Medicine, Microbiology and Computer Science, and the University of Pennsylvania Institute for Strategic Threat Analysis and Response, University of Pennsylvania, 522 Johnson Pavilion, Philadelphia, PA 19104, USA; 4 EMBL, European Bioinformatics Institute, Wellcome Trust Genome Campus, Hinxton, United Kingdom, CB10 1SD, UK

**Keywords:** Chemogenomics, target identification, druggable genome.

## Abstract

Pandemic, epidemic and endemic infectious diseases are united by a common problem: how do we rapidly and cost-effectively identify potential pharmacological interventions to treat infections? Given the large number of emerging and neglected infectious diseases and the fact that they disproportionately afflict the poorest members of the global society, new ways of thinking are required to develop high productivity discovery systems that can be applied to a large number of pathogens. The growing availability of parasite genome data provides the basis for developing methods to prioritize, *a priori* potential drug targets and analyze the pharmacological landscape of an infectious disease. Thus the overall objective of infectious disease informatics is to enable the rapid generation of plausible, novel medical hypotheses of test-able pharmacological experiments, by uncovering undiscovered relationships in the wealth of biomedical literature and databases that were collected for other purposes. In particular our goal is to identify potential drug targets present in a pathogen genome and prioritize which pharmacological experiments are most likely to discover drug-like lead compounds rapidly against a pathogen (*i.e*. which specific compounds and drug targets should be screened, in which assays and where they can be sourced). An integral part of the challenge is the development and integration of methods to predict druggability, essentiality, synthetic lethality and polypharmocology in pathogen genomes, while simultaneously integrating the inevitable issues of chemical tractability and the potential for acquired drug resistance from the start.

## 1. INTRODUCTION

The World Health Organisation estimates that one in six of the world’s population suffers from one or more neglected infectious diseases such as onchocerciasis, trypanosomiasis, lymphatic filariasis, schistosomiasis, soil-transmitted helminthiasis, and blinding trachoma. These are the same poor populations that are at risk from the major infectious disease burdens of malaria, tuberculosis and human immunodeficiency virus [1]. Deaths from infectious diseases occur disproportionately in the developing world, where they are the biggest killer of children and young adults. Communicable diseases account for 50% of the disease burden of the developing countries, which represent 4.8 billion people, 80% of the world population. Yet despite this disease burden only thirteen new drugs were approved between 1975 and 1999 for the tropical diseases [2]. The developed world cannot be complacent about infectious diseases within its own borders. Only one new class of antibiotics for gram-positive bacteria, the oxazolidinones, has been approved in the past four decades [3]. The prospect of new antibiotics against gram-negative bacteria is even bleaker with few drugs against this class of pathogen currently in clinical development [4]. Globally, nearly 340 infectious diseases are reported to have emerged between 1940 and 2004, including many drug-resistant strains of pathogens [5]. The emerging infectious diseases also present a danger to the health and security of the populations of the OECD countries, whether it is the rise of drug resistant pathogens, the threat of a global pandemic or the possibility of a bioterrorist attack.

Infectious diseases pose different threats to different constituencies and thus are often treated as separate problems by governments, businesses, philanthropies and research funders: neglected diseases are treated as global health problems; emerging and pandemic diseases as public health issues; and bio-defence as a national security issue. However, all of these diseases are united by a common problem: how do we rapidly and cost-effectively identify potential pharmacological interventions to treat infections? Given the large number of emerging and neglected infectious diseases and the fact that they often affect the poorest members of society, a general system is required that can be flexibly applied to a larger number of diverse organisms. Compounding the problem of research for new drugs for neglected and emerging infectious diseases is the current productivity paradox in the pharmaceutical industry: as our biomedical knowledge increases we are simultaneously witnessing a decline in the number of new drugs being approved, combined with an economically unsustainable rise in costs. The year 2007 saw the number of new drugs approved for sale drop to 16 new molecular entities (NMEs), its lowest level since 1983 [6] and little increase of new drug approvals in 2008 [7]. Translating new data and knowledge into new therapies is the challenge at the heart of this paradox. New, more cost-effective and efficient methods of drug discovery are urgently required if we are to tackle the multiple global health challenges of emerging and neglected infectious diseases for which there is relatively little basic science investment.

The advent of high-throughput genome sequencing technology offers the possibility that the genome sequence of an emerging pathogen can quickly be determined soon after its identification. However our ability to exploit a pathogen’s genome information, with the goal of identifying potential therapies for testing, is still measured in the order of years, judging by the lack of progress in infectious disease therapies derived from genetic analysis [8].

## 2. NEED FOR INFECTIOUS DISEASE INFORMATICS

The magnitude of the threat of the infectious diseases has resulted in recent calls for global efforts to counter the threat. In particular the application of informatics has been recognised to aid generation, application and management of information and intellectual property to contribute to innovation and promote public health [9-11]. The Sixty-First World Health Assembly’s report of the Intergovernmental Working Group on Public Health on a Global Strategy and Plan of Action on Public Health, Innovation and Intellectual Property (WHA61.21) [9] section 35, 5.1 calls for:

36 (5.1) supporting information sharing and capacity building in the application and management of intellectual property with respect to health related innovation and the promotion of public health in developing countries.

(c) facilitate widespread access to, and promote further development of, including, if necessary, compiling, maintaining and updating, user-friendly global databases which contain public information on the administrative status of health-related patents, including supporting the existing efforts for determining the patent status of health products, in order to strengthen national capacities for analysis of the information contained in those databases, and improve the quality of patents.

Furthermore, Rubin and co-workers at the University of Pennsylvania Institute for Strategic Threat Analysis and Response have called for a ‘Comprehensive International Compact for Infectious Diseases’ [10, 11] to ensure coordinated global research and development efforts are directed to ensuring we have sufficient drugs and preparedness to tackle the present and emerging threat of infectious diseases. The objective of the Compact is to minimize the impact of infectious diseases on national and international health, maximize social and economic development and enhance international security by creating a coordinated, global approach to the problems. In order to undertake this objective effectively, Rubin proposes the legal framework of a Compact to develop a comprehensive agreement between governments powers, the scientific community, the private sector and other stakeholders that will limit and control known, newly discovered or deliberately created infectious diseases. The four missions of the Compact are

1. Establish, maintain and monitor a shared international data and knowledge base for infectious diseases, including but not limited to bio-surveillance information, relevant pharmaceutical and basic research data and suites of services and skills.
2. Establish, implement, maintain and monitor a network of international basic science research centers that will support fundamental investigations into the pathophysiology of certain microbial threats to global health.
3. Expand capabilities for the production of vaccines and therapeutics expressly for emerging and re-emerging infections.
4. Establish, implement, maintain and monitor international standards for best laboratory and regulatory practices.

The infectious disease informatics strategy we discuss below is a response to both the aims of Mission I of the International Compact for Infectious Diseases and the Section 36:5.1(c) of the Sixty-first World Health Assembly’s resolution WHA61.21 on Global Strategy and Plan of Action on Public Health, Innovation and Intellectual Property to ensure the effective use of information to encourage innovation in discovery and development of new infective disease medicines.

## 3. DRUG DISCOVERY STRATEGIES

The multiple challenges of endemic, pandemic and epidemic neglected and emerging infectious diseases calls for a global, systematic approach to accelerating our current efforts at discovery of pharmacological agents against these afflictions [9-12]. The rapid advances in genome sequence technology and informatics provide us the tools to devise a strategy to systematically exploit the wealth of information continuously being generated by the global biomedical enterprise for other purposes, and apply it to the search for new agents for infectious diseases. Thus the overall objective of this review is to illustrate the types of systems and general methodologies that could enable the rapid generation of plausible *and *testable medical hypotheses for pharmacological experiments by uncovering as yet undiscovered and unexplored relationships in the biomedical-related databases and literature corpus. In particular, our goal is to identify potential drug targets within pathogen genomes [13] and host genetic factors [14, 15] and prioritize which pharmacological experiments are most likely to rapidly discover drug-like lead compounds against a pathogen (*i.e.* which specific compounds and drug targets should be screened, in which assays and where they can be sourced or undertaken).

### 3.1. Rethinking Genome-Based Drug Discovery

The growing number of available genome sequences of human pathogen allows, for the first time, the rational prioritization of all potential drug targets for a wide range of emerging and neglected infectious diseases. The first generation of genome-based drug discovery projects have had relatively little success, to date, especially in the field of anti-bacterials [8,16]. The major drawback of earlier approaches was that despite significant upfront investment in understanding the basic biology and target validation, to build confidence-in-rationale in the molecular target, there is a high risk that drug screening efforts will yield very little. Payne *et al.* recently described the challenges of an industrial genome-lead high throughput screening strategy to discover novel anti-bacterials [8]. Over 7 years GSK invested in the target validation of over 300 bacterial targets and showed 160 of them to be genetically essential. Seventy of the essential targets were screened against GSK’s corporate compound collection in high throughout screens. The results were disappointing. Only 16 of the 70 HTS run gave hits and only 5 resulted in the discovery of lead compounds against (peptide deformylase (PDF), enoyl-acyl carrier protein reductase (FabI), 3-ketoacyl-acyl carrier protein III (FabH), methionyl tRNA synthetase (MetRS) and phenylalanyl-tRNA synthetase (PheRS) targets). Following initial efforts in lead optimization only one lead series for PheRS were still being pursued at the time of publication. The unsustainable failure rate genome-lead anti-infective drug discovery calls for a re-evaluation of the assumptions behind the strategy.

#### 3.1.1. Chemogenomics

One of the key reasons for the failure of the first generation of anti-infective genomics-lead drug discovery campaigns was dominance of biological over chemical considerations at the target selection stage. Chemical considerations, such as tractability as a way of assessing a target’s ‘druggability’ and the diversity or appropriateness of the chemical space covered by compound libraries being screened against novel targets, are vital factors in improving the likelihood of success of screening campaigns. The ‘druggability’ of a molecular target, such as a protein, is its inherent ability to be modulated by a high affinity, ‘drug-like’ small molecule [17-21]. There are strong evolutionary arguments why proteins have evolved exquisite molecular recognition capabilities to avoid unwanted functional disruption in the vast sea of small molecule metabolites in which they exist. Current estimates, from analysis of the pharmaceutical industry screening data, suggest that only approximately 15% of proteins expressed by an organism’s genome have any inferred evidence of being potentially modulated by drug-like compounds [13, 17, 22]. Additionally druggability is an attribute that is likely to be independent of lethality. Many genome-scale comprehensive knock-out studies in model organisms have consistently identified around 19% of genes to be individually essential. Thus targets that are both lethal and druggable represent an intersect that is less than 3% of the proteins expressed in a genome (assuming lethality and druggability are not correlated factors). When selectivity and the often required broad activity spectrum across many genomes is taken into account (as is the case with anti-bacterial target hunting) then even this small percentage of suitable proteins in a genome, decreases further still.

Despite the fact that our knowledge of observed attributes of the vast majority of pathogen proteins is limited or missing, it is possible to develop methods to prioritize potential drug targets from a pathogen genome *a priori* by inference from the collective wealth of bio-pharmacology knowledge available, such as genome sequences, model organism knock-outs, protein structures, medicinal chemistry structure-activity data and literature abstracts [13, 23-26]. The strategy of exploiting the wealth of knowledge of drug targets and associated compound properties is known as chemogenomics [27-30]. Proteins deemed to be potential drug targets would be those which are known or predicted to have a high probability of finding a drug-like chemical lead, and are known or are inferred to be essential to the pathogen for which selective drugs, non-toxic to humans could be developed. For example druggability can be assessed *a priori* from protein sequence and protein structures using large-scale, chemogenomics databases [17, 31] and protein structure binding site analysis [18, 19, 21]. In addition to the published data on genes known to be essential for certain pathogens, lethality data of which genes are predicted to be essential can either be inferred from orthologues of large-scale model organism knock-out studies [13, 23, 32] or predicted by network analysis of metabolic or reconstructed biochemical networks [33-38]. Importantly essential genes do not have to be unique to a parasitic organism to be drug targets. Molecular differences in drug binding sites, identified through protein structure can be exploited by drug design to achieve selectivity and therapeutic index. For example, the clinically useful drug trimethoprim and its derivatives inhibit bacterial dihydrofolate reductase (DHFR) but not the related essential human enzyme. Selectivity between homologous human and parasite proteins can be calculated by analysis of the sequence of binding sites of protein models at genome-scale [13, 39, 40]. Toxicity, or other undesired pharmacology that may result from binding to a host genome orthologue, can often be designed out.

Furthermore, not only is it important to prioritize potential drug targets based on a set of criteria and known and inferred attributes, also it is also necessary to identify potential pharmacological experiments to undertake - by providing details of specific compounds to test, assay protocols, as well as the names of relevant experts and materials, based on an objective analysis of all the available biomedical information. The availability of pathogen genome data therefore provides the basis for developing methods to prioritize, *a priori,* the potential drug target and pharmacological landscape of an infectious disease. This has not been the case until now.

Traditionally whole organism screening against specific compound collections has been the primary method of anti-infective drug discovery. The principle drawback of screening chemically diverse libraries with no knowledge of specific protein is the ratio of the global *in vivo* screening capacity to the total number of potentially available compounds. In contrast, chemogenomic-based drug discovery against specific targets is designed to test a large number of compounds in molecular biochemical or binding assays in order to triage down to a small number of compounds, with pre-selected drug-like properties, with known or predicted *in vitro* effectiveness against specific targets. The most promising compounds can then be tested in whole organism (*in vitro*) and in animal models (*in vivo)*. Thus chemogenome-based strategies enable a far greater number of compounds with a higher *a priori* probability of being active *in vivo*. This is especially important because there is a limited capacity for performing *in vivo* screening assays, which is a particularly acute problem in the area of the neglected parasitic diseases. There have been tentative developments to apply a chemogenomics approach to parasite genomes, the most advanced of which is the TDR Targets Database, sponsored by the World Health Organisation Special Programme for Research and Training in Tropical Diseases (TDR) (http://TDRtargets.org), where a limited amount of druggability information has been calculated and disclosed for *Mycobacterium tuberculosis (tuberculosis)*, *Plasmodium falciparum* (Malaria), *Trypanosoma brucei* (Human African Trypanosomisas), *Trypanosoma cruzi *(Chagas' disease)* and Leishmania major *(Leishmaniasis) ^13^. Genome-scale drug target prioritization strategies have also been applied by other groups to *Mycobacterium tuberculosis* [25], *Brugia malayi* (lymphatic filariasis) [23] and *Schistosoma mansoni* (schistosomiasis) [26]. However, a significant amount of further development is need to improve the utility of the chemogenomics data and druggability prioritisation methods used in the first attempts at whole genome drug target rankings, not only for selected tropical disease pathogens but, potentially, for all human pathogens. The issue of prioritization of drug targets from pathogen genomes is vital if we wish to maximise the limited drug discovery resources available to neglected diseases, where the global drug discovery portfolio against some of the neglected diseases may only be handful of credible screening projects.

#### 3.1.2. Polypharmacology

A major aim of the first generation of genome-based drug discovery projects was to identify single gene products/proteins that are essential when deleted. This focus on single, essential proteins is limited for two reasons. First, the downstream difficulty and low inherent likelihood of discovering small molecule leads has often only been considered after significant investment in biology (hence the argument for chemistry-led approaches, as discussed above). Secondly, if a drug is discovered, a single amino acid mutation is often enough to confer drug resistance. However, many effective antibiotics act by targeting multiple proteins simultaneously rather than individual proteins. In contrast to the ‘single target approach’, multi-drug combination therapies are the main strategy to reduce the development of drug resistance in many anti-infective regimes, such as current anti-HIV and anti-tuberculosis therapies. By attacking multiple, mutually exclusive drug binding sites in a pathogen’s genome the emergence of drug resistance can be delayed as the probability of simultaneously developing multiple mutations, even in a single organism can be less than the pathogen population size multiplied by the rate of mutation. However, the ability of multi-drug combination therapies to delay the emergence of drug resistance is dependent on the continuous presence of optimal drug concentrations, which in turn is dependent on patient compliance, which can be challenging when a patient is faced with adhering to a cocktail of drugs with complex dosing schedules and varying pharmacokinetics. Reduction in the levels of just one drug can result in drug resistance emerging. Reviews of the modes-of-action of the current pharmacopeia of anti-bacterials reveals the majority of effective antibiotics act by targeting multiple proteins simultaneously rather than individual proteins [41-43]. By searching for single essential targets the first generation of genome-lead anti-bacterial drug discovery projects failed to rediscover many of the known antibiotic drug targets. For example, the anti-bacterial action of β-lactams are dependent on the inhibition of at least two of the multiple penicillin-binding proteins (PBPs) since multiple PBPs can be deleted with no effect on phenotype [44]. Similarly, fluoroquinolone antibiotics are dually targeted inhibiting the topoisomerase proteins ParC and GyrA 58 [45]. D-cycloserine acts on four targets, through inhibition of both pairs of alanine racemase and D-ala-D-ala ligase. Likewise, fosfomycin overcomes the redundancy of UDP-N-acetlyglucosamine enolpyruvyl transferases by inhibiting them both. This feature of multiple genes coding for a core biological function can be seen in the context of the evolution of a ‘robust system’, relatively immune to external influence and control – precisely what is required for a pathogen to co-exist with hosts capable of mounting a response to infection.

Systematic experiments with dual knock-outs in model systems have shown that, whilst the deletion of two genes in isolation may show no effect, the simultaneous deletion of two genes can lead to ‘synthetic lethality’ or ‘synthetic sickness’. Therefore if we wish to design single drugs that limit drug resistance we could consider the development of methods to identify and prioritize combinations of targets that can be inhibited by the same compound and are either all essential individually or in combination (*i.e.,*synthetic lethality) [41, 42, 46]. Based on the examples above, common functions encoded by paralogous genes is one such simple prioritisation strategy. By targeting two or more essential genes with a single chemical agent, the ability to delay drug resistance is designed into the target discovery strategy from the start. Given the failure of current genome-based strategies for discovering new antibacterial drugs, learning the lessons of the previous successful generation of antibacterial drugs may encourage the development of anti-infective drug discovery strategies with an inherently greater chance at delaying the emergence of drug resistance. A strategy of polypharmacology – compounds that act *via* multiple proteins – may therefore increase the opportunity space by increasing the number of essential target systems [47] and delaying the emergence of drug resistance [42, 48]. The polypharmcology strategy can also be applied to the design of single drugs that could limit the evolution of drug resistance, if we developed methods to search and prioritize which combinations of targets can be inhibited by the same drug and are both individually essential. Thus we are both expanding the range of proteins we may consider and prioritize as drug targets but also pragmatically focusing on exploiting the wealth of current chemical and pharmacology information.

Informatics methods for assessing selectivity have also a complementary application in the prediction of polypharmacology. In order to devise methods to predict polypharmacology it is useful to classify different types of polypharmacology behaviours. Therefore we propose classifying polypharmacology into three classes to assist algorithm development.

##### 3.1.2.1. Type I Polypharmacology

Refers to the binding of a compound to related homologous targets. The promiscuous binding of many inhibitors between related protein kinases is an example of this type of polypharmacology. Structure-based binding site sequence/structure alignments are therefore a useful tool for assessing selectivity across a gene family.

##### 3.1.2.2. Type II Polypharmacology

Refers to a compound binding to proteins that are non-homologous at the sequence level but share a common endogenous substrate or ligand. Metabolic and pathway databases are useful data sources for identifying potential candidates for Type II polypharmacology.

##### 3.1.2.3. Type III Polypharmacology

Refers to the binding of a compound to targets that are non-homologous at the sequence nor share common endogenous substrate or ligand. There may indeed exist unexpected commonalities in the topology of physico-chemical characteristics of two apparently unrelated binding sites by chance. Several chemoinformatics methods of assessing chemical structure similarity show promise in prediction of Type III Polypharmacology between unexpected targets [31, 49].

#### 3.1.3. Identifying Broad Spectrum Targets

In addition to the polypharmacology behaviour of agents against multiple proteins within a pathogen one can also consider polypharmacology of agents between organisms. A compound that is predicted and observed to bind to essential targets across a number of pathogens, yet be selective against a human orthologue may have the potential to be developed into a drug with a broad anti-parasitic spectrum to reduce the burden of patients suffering from multiple infections, in particular the common experience of poly-parasitism in the developing world [50]. Although the chances of such discoveries may be small, initiating systematic search methods for polypharmacology opportunities across multiple genomes may identify several such opportunities. Large-scale comparative chemogenomics can systematically identify cross-species opportunities for anti-infective drug discovery by mapping the landscape of all putative drug targets, providing a common framework on which to focus the global efforts in tropical disease research. Therefore comparative chemogenomics and essentiality analyses across a large number of pathogen genomes can provide valuable information, especially in identifying common drug targets between pathogens, specifically searching for drugs against poly-parasitism or identifying the closest pathogen proteins most likely to bind a specific set of compounds.

### 3.2. Indications Discovery: Reprofiling of Drugs and Targets

In contrast to phenotypic-based screening or genomics-led approaches to drug discovery there are emerging discovery strategies that may be beneficial to anti-infectives drug discovery. One such strategy is indication discovery [51], also known as drug reprofiling or drug repurposing [52, 53]. The history of medicine is replete with examples of serendipity: compounds, which were originally developed for one disease being subsequently found to be beneficial against another disorder. Far from being rare, the number of therapies being marketed for new indications per annum is rising and about equal to the number of new therapies arising from new compounds with novel mechanisms of action reaching the market each year [54] . One of the most famous examples of serendipity in medicine is Alexander Fleming’s discovery of penicillin. However, the history of penicillin also contains another lesson for drug discovery: buried in the scientific literature are several independent experiments on the anti-bacterial effects of Penicillium [55]: Billroth in the 1860s by Lister, Tyndall, Roberts, Pasteur and Joubert in the 1870s and by Duchesne in 1886 and by Twight and Gratia and Dath in the 1920s. The examples of the repeated discovery of penicillin illustrate what Swanson calls ‘undiscovered public knowledge’ [56]. Indeed, protein targets as well as compounds may find alternative medical utility from their primary disease association [51, 57]. Ironically, the sheer wealth of biomedical information may itself be an impediment to progress. The exponential growth of published biomedical knowledge creates the paradox where the individual scientist or clinician knows a diminishing fraction of all knowledge in the field. The drawback of search engines such as Pubmed or Google Scholar is that they only allow one to find what one is searching for, whereas the lesson of serendipity in science is that a development in any one field may be of value in generating novel hypothesis in another, apparently unrelated field [58]. Thus serendipity could be mimicked by developing systems that mine the mass of apparently unconnected biomedical information to propose and score new medical hypotheses for new treatments for unmet medical needs [57].

Given the rapid rise of new knowledge, information systems that automatically search for associations between proteins, compounds and diseases could have immense impact in the biomedical sciences, in particular as a cost effective method of discovering pharmacological agents against neglected and emerging infectious diseases. In the field of anti-infectives there is a growing record of parasite drug targets that are orthologues of human drug targets. For example, cholesterol biosynthesis inhibitors of human squalene synthase have been demonstrated to block *Staphylococcus aureus* virulence by the inhibition of the structural homolog *S.aureus* dehydrosqualene synthase [59]. Likewise the chemical inhibitors of the *Plasmodium falciparum* orthologues of the human anti-cancer target methionine aminopeptidase 1b possess antimalarial activity [60, 61]. The folate pathway has proved a rich target for drug treatment not only for anti-cancer and rheumatoid arthritis but also for several parasites include *P. falciparum* and the *T. brucei*. The advantage of finding new uses for existing compounds and drug targets is the significant saving in both cost and time compared to the *de novo* discovery and development of a new chemical or biological entity, acting *via* an unprecedented drug target.

## 4. DISCOVERY INFORMATICS FRAMEWORK

In order to systematically map out the potential pharmacological landscape for a pathogen a framework is required that can guide the development of informatics services that would enable the range of diverse drug discovery strategies outlined above. If our goal is to systematically infer the attributes of pathogen proteins relevant to drug hunting and prioritize those attributes by practical considerations of drug discovery then we propose the required informatics infrastructure will need to contain three main overlapping areas:

1. Druggability
2. Selectivity
3. Efficacy

Ultimately a potential pathogenic drug target is identified by its attributes describing the likelihood of discovering small molecule modulators and in the confidence we assign to the prediction of those attributes.

### 4.1. Druggability

The ‘druggability’ of a target encompasses the chemical space associated with a protein, which in turn is determined by the physico-chemistry and molecular architecture of the binding sites of interaction [17-19, 21]. A number of investigators have used the physico-chemical basis of molecular interactions to predict druggability based on protein structures [18, 19, 21, 62-64]. Since exact structural information is not known for the vast majority of pathogen proteins, sequence based similarity methods can be applied to identify pathogen proteins that are homologous to proteins that are known to bind small molecule, drug-like ligands found in the chemogenomics database such as ChEMBL (www.ebi.ac.uk/chembl). However, the limitations of inferring druggability by solely sequence-based methods include the observation that many druggable protein families may share little overall sequence similarity yet still maintain a common druggable binding site, and that the drug binding site may be composed of elements from several constituent proteins in a complex, with each component needing to be present for the binding site to exist. Moreover many drug targets have multiple ligand binding sites, which in turn can have different modes of action (*e.g.* substrate competitive, allosteric) and that each of these binding sites exhibit different degrees of druggability and bind ligands from different areas of chemical space. Drug binding sites are far more diverse than defined enzymatic active sites within single protein domain. Indeed several known drug-binding sites occur at the interfaces between domains and protein complexes. The creation of the binding site itself may also be a temporal process that depends upon post-translational modification, induced fit or an allosteric cooperativity between other events in the protein complex. Thus one approach to inferring druggability across a highly diverse sequence space is to construct an ontology that defines the molecular interactions between components of binding sites and then use this ontology as a platform for sequence based analysis. However, our goal is not only to identify which pathogen or host proteins are likely to be druggable but also to identify specific compounds and areas of chemical space which have the highest likelihood of being active against the putative drug targets of interest. Prioritized compounds can then be sourced from commercial or proprietary compound collections for screening in appropriate assays.

Until recently the vast amount of pharmacological data linking chemical structures to biological activities have not been available in computationally accessible formats. The vast majority of biological activity data on chemical structures resides within the informatics systems of the large pharmaceutical companies. Whilst a fraction of the more useful screening data in these sources has previously been publicly disclosed in the form of patents or (less often) in journal articles, the information is not easily accessible for large-scale machine learning. In order to mine the wealth of biologically-active chemical information that is present in patents and medicinal chemistry journals, the information needs to be extracted, standardized, normalised and cross-validated. Recently our ability to access and mine these data, outside of the pharmaceutical industry, has improved considerably. Firstly, the National Institutes of Health (NIH) Roadmap initiative has lead to the public deposition of large amounts of novel screening information, from the NIH Molecular Libraries and Screening Centers Program in the PubChem repository. Secondly, the Wellcome Trust has made a major £4.7 million investment at the EMBL European Bioinformatics Institute (EMBL-EBI, Cambridge UK) to place a large amount of curated medicinal chemistry data in the public domain, in the form of the ChEMBL databases. The ChEMBL database of chemical structure-activity relationship information comprises of data extract from the medicinal chemistry literature since 1980*. *This database represents a comprehensively annotated chemogenomics resource of approximately 450,000 synthetically tractable bioactive compounds and covering around 5,700 curated targets which have been abstracted from over 26,000 articles from the primary literature, most notably *Journal of Medicinal Chemistry* (1980-2009) and *Bioorganic Medicinal Chemistry Letters* (1990-2009). These two journal contain 90% of the published medicinal chemistry structure-activity data according to a recent study by AstraZeneca (P. Leeson, pers. comm.) The ChEMBL databases also contain a curated set of approved drugs and their targets, with enhanced and complementary annotation compared to other public resources such as Drugbank [65]. These databases provides an essential data source for inferring small molecule druggability for pathogen proteins. Thirdly, advances in text mining have enabled the development of chemical name recognition software that can be used to extract chemical names from the vast amount of patent information that exists globally. IBM, for example, in collaboration with a number of pharmaceutical companies, have trained a chemical extraction engine to recognise the nuances and common errors that often occur in the text of patents and patent applications and have used this technology to extract over 12 million unique chemical structures from the patent corpus of over 12 million patent documents and over 18 million Medline abstracts, titles and annotations. Using IBM’s Blue Gene computer this database of chemical structures extracted from patents can be updated daily. Routinely the system annotates over 100,000 documents per month but is capable of annotating around 1 billion pages in approximately 3 hours, if necessary. This wealth of chemical data, that can now be linked to biological activity with varying degrees of confidence, provides the basis for considering that it may now be possible to link suitable and accessible chemistry to pathogen drug targets in a systematic and timely fashion using a range of virtual screening tools, including: 1-dimensionals fingerprint based similarity tools such as Bayesian activity modelling [31, 66-68] and Similarity Ensemble Approach [49, 69]; 2-dimensional graph-based and pharmacophore matching tools [70, 71]; and 3-dimensional structure-based docking tools, where protein structural data is available (from direct x-ray crystal structural data or inferred *via *comparative modelling) [72].

### 4.2. Selectivity

A second key attribute to identifying and prioritizing pathogen drug targets is a selective therapeutic index (TI) between the parasite and the host. Conventionally, the perceived requirement for absolute selectivity over host functionality has typically led to the strategy of selectively searching for pathogen targets that are not present in the host. The absence of an orthologue from the host of a pathogen drug target is a valid assumption, but it does rule out many core essential genes that are common between diverse species. However, selectivity between a drug target that is common between the host and pathogen can still be obtained by (i) exploiting amino acid differences in the binding site (binding site selectivity), or (ii) by selecting compounds which exhibit different kinetic off-rates between the host and pathogen target (kinetic selectivity) or (iii) by a difference in biological importance of the target to each organism (biological selectivity) or (iv) by designing (or discovering) a compound that is differentially metabolised from a prodrug or into a inactive metabolite between host and pathogen (metabolic selectivity) or (v) by differences in transport and excretion (for example, active uptake). An important aid in understanding binding site selectivity in particular is comparative modelling and binding site sequence alignments. Sali’s group at UCSF has developed a proteome scale modelling workflow that has been applied to a number of human pathogen genomes [40]. The output models have been made available to both the TDR Targets database [13] and the Tropical Disease Initiative’s kernel [39, 73].

### 4.3. Efficacy

The third key attribute necessary to prioritize a pathogen drug target is its relevance or functional linkage to the control of the disease pathology, or what we shall call its ‘efficacy’: the biological quality of the drug target. We define a target’s efficacy as the effect its modulation has on the infectious disease state. In short, it is an estimate of the capacity for a compound binding to a target to decrease the scale or severity of the infection. Whilst the concepts of druggability and selectivity are ultimately physico-chemically based, the concept of ‘efficacy’ is context-based, dependent on the holistic functioning of the host-pathogen system. The continuum of efficacy stretches across essential genes, synthetically lethal gene combinations, virulence factors and other host factors. To achieve the desired lethal or static effect different degrees of inhibition may be required. For example, genetic knockouts result in the complete ablation of both the enzymatic and structural functions of a protein. If the essential properties of the protein is due to it structural role, no enzyme active site inhibitor, no matter how potent, can mimic that deletion. The exact mimicking of a genetic knockout by a chemical may be practically impossible. To achieve the required functional suppression an irreversible covalent inhibitor, or very slow inhibitor k_(off)_ kinetics may be required to provide insurmountable inhibition [74, 75]. The issue of insurmountable inhibition maybe particularly important if the target is extracellular where mass action effects are unfavourable as the ligand may diffuse off, if there is a high concentration of competing substrate or if a high level of inhibition is required to perturb the organism [42]. Predicting the effects of a partial degree of perturbation or even complete deletion, of a particular protein on a pathogen-host disease state is one of the most difficult scientific challenge facing the infectious disease community. However there are a growing number of experimental and computational methods than can be employed to tackle this challenge. In addition to published data on genes known to be essential that exist for some pathogens, lethality data of which genes are predicted to be essential can either be inferred from orthologues of large-scale model organism knock-out studies [13, 23, 32] or predicted by network analysis of metabolic or reconstructed biochemical networks [33-38]. One hypothesis that appears correlated with the lethality of single gene deletions and synthetic lethality of double knock-outs is a topological one - the ‘missing alternative’ in metabolic networks [76, 77]. Essentiality and synthetic lethality can be considered as emergent properties that result from biochemical network wiring and dynamic rewiring. The ‘missing alternative’ hypothesis suggests synthetically lethal genes function in parallel or in compensating pathways. 

In addition to the large-scale knock out studies in pathogen genomes, genome-wide RNA interference is increasingly being used to identify host factors critical for pathogen infection, such as HIV-1 [14], the flavivruses [78], West Nile virus [15] and Dengue virus [79]. The discovery of leads against human host factors also benefits from the systematic chemogenomics analyses outlined above. Indeed, the recent approval of maraviroc [80], a potent, selective drug targeting the human CCR5 chemokine receptor, a major co-receptor for HIV-1, illustrates the value of targeting essential host factors.

## 5. CONCLUSIONS

A survey of the global pharmacology space reveals that with our current chemogenomics sources we can identify drug-like chemical leads for *ca* 1500 protein targets [31]. Using a variety of established and developmental chemoinformatics and bioinformatics techniques we can potentially link the known biologically active chemical space to a wider range of proteins to identify putative and focussed sets of compounds for screening. The informatics strategy outlined here holds the promise of making significant impact on infectious disease drug discovery and bio-defence strategies against emerging pathogens. The information-based strategy is global, systematic and scalable and can potentially be applied to analyze the genomes of protozoa, helminths, fungi, bacteria and viruses. The strategy we have outlined is designed to rationally identify targets with a high chance of finding a good chemical starting point, from which selective drugs, that are non-toxic to humans, could be developed, and also extends the range of current targets for consideration to include synthetically lethal and polypharmacology targets. The scalability of the strategy and the increasingly ‘open’ nature of scientific data indicates that its chance of success increases as it is applied to a wider range of pathogens and integrates data from a wider range of information sources.

The strategy outlined here could greatly enable the cross-fertilization of ideas between independent groups and across geography. A number of collaborations could be spurred by alerting scientists who are working on specific compounds or protein in one area that their research could be beneficial to another group working on an infectious disease, elsewhere. By actively and systematically searching for connections between genomes, compounds, assays and drug targets the limited resources available to neglected and emerging diseases could be expanded as they would benefit from the greater, global biomedical enterprise by enabling the deliberate re-use of knowledge and provide greater returns on funding agency investments.
